# Ionized Magnesium: Interpretation and Interest in Atrial Fibrillation

**DOI:** 10.3390/nu15010236

**Published:** 2023-01-03

**Authors:** Jean-Baptiste Bouillon-Minois, Louisa Khaled, Florence Vitte, Ludovic Miraillet, Romain Eschalier, Matthieu Jabaudon, Vincent Sapin, Lucas Derault, Samy Kahouadji, Marina Brailova, Julie Durif, Jeannot Schmidt, Fares Moustafa, Bruno Pereira, Emmanuel Futier, Damien Bouvier

**Affiliations:** 1 Emergency Department, Université Clermont Auvergne, CNRS, LaPSCo, Physiological and Psychosocial Stress, CHU Clermont-Ferrand, 63000 Clermont-Ferrand, France; 2 Anesthesiology and Critical Care Department, CHU Clermont-Ferrand, 63000 Clermont-Ferrand, France; 3 Service des Urgences, CHU Clermont-Ferrand, 63000 Clermont-Ferrand, France; 4 Cardiology Department, CHU Clermont-Ferrand, 63000 Clermont-Ferrand, France; 5 Department of Anesthesiology, Critical Care and Perioperative Medicine, CHU Clermont-Ferrand, 63000 Clermont-Ferrand, France; 6 Department of Medical Biochemistry and Molecular Genetics, CHU Clermont-Ferrand, 63000 Clermont-Ferrand, France; 7 Biochemistry and Molecular Genetic Department, CHU Clermont-Ferrand, 63000 Clermont-Ferrand, France; 8 Biostatistics Unit (DRCI), CHU Clermont-Ferrand, 63000 Clermont-Ferrand, France

**Keywords:** ionized magnesium, atrial fibrillation, emergency medicine, critical care, cardiology

## Abstract

Background: Magnesium (Mg) is often used to manage de novo atrial fibrillation (AF) in the emergency department (ED) and intensive care unit (ICU). Point of care measurement of ionized magnesium (iMg) allows a rapid identification of patients with impaired magnesium status, however, unlike ionized calcium, the interpretation of iMg is not entirely understood. Thus, we evaluated iMg reference values, correlation between iMg and plasmatic magnesium (pMg), and the impact of pH and albumin variations on iMg levels. Secondary objectives were to assess the incidence of hypomagnesemia in de novo AF. Methods: A total of 236 emergency department and intensive care unit patients with de novo AF, and 198 control patients were included. Reference values were determined in the control population. Correlation and concordance between iMg and pMg were studied using calcium (ionized and plasmatic) as a control in the whole study population. The impact of albumin and pH was assessed in the discordant iMg and pMg values. Lastly, we assessed the incidence of ionized hypomagnesemia (hypoMg) among de novo AF. Results: The reference range values established in our study for iMg were: 0.48–0.65 mmol/L (the manufacturers were: 0.45–0.60 mmol/L). A strong correlation was observed between pMg and iMg (r = 0.85), but, unlike for calcium values, there was no significant impact of pH and albumin in iMg/pMg interpretation. The incidence of hypoMg among de novo AF patients was 8.5% (12.7% using our ranges). When using our ranges, we found a significant link (*p* = 0.01) between hyopMg and hypokalemia. Conclusion: We highlight the need for more accurate reference range values of iMg. Furthermore, our results suggest that blood Mg content is not identical to that of calcium. The incidence of ionized hypomagnesemia among de novo AF patients in our study is 8.5%.

## 1. Introduction

Magnesium (Mg) is one of the most important cations in the human body [1,2]. It is concentrated in the intracellular compartment, especially in bones—with a strong relationship with calcium (Ca) and phosphate—but also in muscles and soft tissues [2,3,4,5]. Plasma magnesium (pMg) represents 2% of the total body Mg [6,7,8], and around 70% of pMg is ionized (iMg) and 30% is bound to albumin [6]. As a divalent cation, Mg content and physiology are often compared to that of calcium (ionized (iCa) and plasmatic (pCa)) but are much less studied [2]. For instance, in the interpretation of pMg values (the only indicator of Mg status), the impact of albumin and pH variations is not completely understood [9,10]. A recent international expert group, suggests that the lower limit for serum Mg should be increased to 0.85 mmol/L [11].

The main clinically relevant manifestation of dysregulation of Mg homeostasis is hypomagnesemia (hypoMg), concerning 2% of the general population [2]. It is usually defined as pMg under 0.75 mmol/L [7], or iMg under 0.45 mmol/L [12]. The symptoms of hypoMg include weakness, nausea, cramps, muscle contractions, seizures, and arrhythmia [9]. A profound Mg depletion also impacts the homeostasis of potassium and calcium and can cause hypokalemia and hypocalcemia [7]. Dysregulation of Mg homeostasis symptoms is linked to the key role played by Mg in many physiological functions. As a cofactor, Mg is involved in over 600 enzymatic reactions [13], including the functioning of the cardiovascular system via calcium ATPases and especially for modulation of cardiac potential action [14]. Through phosphorylation, Mg inhibits L-type calcium channels, exerting a protective effect on myocardial cells [15]. Furthermore, Na-K ATPase requires Mg to modulate action potential duration. Lastly, Mg’s effect on cardiomyocytes also depends on its ability to compete with calcium for binding sites in proteins, to act as a substrate along with adenosine triphosphate for cardiac calcium ATPases and to alter the affinity of natrium-calcium exchanger [1]. One of the most frequent complications of abnormal potential action is atrial fibrillation (AF).

AF is the most common sustained arrhythmia, with an estimated prevalence of up to 37.6 million cases worldwide, its prevalence increased by 33% during the last 20 years [16,17]. AF is responsible for 600,000 emergency department (ED) admissions in the United States every year [18]. Around a quarter of Intensive Care Unit (ICU) patients have at least one episode of AF [19]. The management of AF is based on the reduction of the ventricular rate. Although not perfect, several drugs are recommended for the management of AF including calcium channel blockers, beta-blockers, and digoxin [20]. The administration of Mg in acute atrial tachyarrhythmia has also been proposed as an alternative or in addition to the usual care [21]. However, the incidence of hypomagnesemia among de novo AF remains unknown.

Point of care measurement of iMg allows a rapid identification of patients with impaired magnesium status, however, unlike iCa, the interpretation of iMg in relation to total magnesemia is not entirely understood. In our study, we evaluated iMg reference values, iMg and pMg correlation, and the impact of pH and albumin variations on iMg levels. The secondary objective was to assess the incidence of hypomagnesemia in AF.

## 2. Materials and Methods

### 2.1. Study Design

We performed a prospective monocentric observational study in the ED, and three different ICUs (general, trauma, and cardiac) of the University Hospital of Clermont-Ferrand, France, between 30 November 2020, and 11 December 2021. The study was approved by the Ethics Committee South-East VI as number 2020/CE65. Patients were informed of their right to express their disagreement regarding the use of their clinical information for research purposes.

The study population consisted of de novo AF patients, diagnosed by an attending physician using a 12-lead electrocardiogram. Exclusion criteria were refusal to participate, medical history of AF, and age < 18 years old. Blood samples were collected for each patient on a heparinized syringe for blood gas analysis and measurement of the ionized magnesium concentration.

A control population (*n* = 198) was constituted of 80 ED and 118 ICU patients without AF, for whom blood samples were collected for routine tests. The inclusion criteria were every patient present in the ED on 30 October or 31 October 2021, or in the ICU between 1 November and 15 November 2021, that already had a blood sample, and the exclusion criteria were patients with AF. Mg reference range was determined in the 80 ED patients, and compared to the manufacturers’, and the prevalence of hypo-iMg was assessed in the whole control population (ED and ICU patients) and compared to that of AF patients.

### 2.2. Data Collection

The data collected at admission were age, sex, body mass index (BMI), CHADSVASC score, medical history of dysthyroidisim, hypertension, medications known to induce hypoMg, sepsis, dehydration, and outcome of the AF episode (resolutive vs non-resolutive). Hyperthyroidism was defined by a TSH < 0.358 mUI/L, or if the patient was known for hyperthyroidism on the case report form (CRF). Dehydration was defined by a diastolic blood pressure < 40 mmHg or systolic blood pressure < 90 mmHg or mean blood pressure < 65 mmHg or plasmatic protein > 80 g/L or if the symptom « hypovolemia » or « dehydration » was validated by the investigator on the CRF. Sepsis was defined if temperature was >38.5 °C or if diagnosed by the physician and reported on CRF. Hypertension was defined by a systolic blood pressure > 180 mmHg or diastolic blood pressure > 100 mmHg or if the patient had a history of high blood pressure or was treated for high blood pressure. Plasma and whole blood results were also collected for iMg, pMg, iCa, pCa, pH, potassium (K), and albumin.

### 2.3. Blood Assays

Whole blood iMg, iCa and pH were determined by direct potentiometry using a Stat Profile^®^ PRIME Plus analyzer from Nova Biomedical (Les Ulis, France). Regarding iMg, the lower detection limit was 0.05 mmol/L and the measuring range extends up to 1.5 mmol/L. Typical within-assay precision was below 2%. The iMg manufacturer’s reference ranges are 0.45–0.60 mmol/L [12]. The reference range values for iCa are 1.09–1.3 mmol/L, and 7.35–7.45 for pH. pMg, pCa, K and albumin measurements were performed using a Vista^®^ analyzer (Siemens, Munich, Germany) following the manufacturer’s recommendations. Correction formulas were used for Ca and Mg: cCa (albumin-corrected calcium) = pCa + 0.025 × (40-albumin) [22] and cMg (albumin-corrected magnesium) = pMg + 0.005 × (40-albumin) [9].

### 2.4. Statistics

Statistical analyses were performed with Stata Software^®^ (v15, College Station, StataCorp, TX, USA). Continuous variables were expressed as the mean and standard deviation (SD) or median [p25; p75] and categorical variables were expressed as number (%). The Gaussian distribution for each ionic variable was assessed by a Shapiro–Wilk test. The first part of this study was performed among our control population, i.e., patients without AF. Secondly, we described our ranges (mean, median, 5%, 95%, p25, p75) of iMg and pMg values, using the control population. We assessed correlation using the Spearman coefficient for iMg, pMg, and cMg, interpreted as follows: [0; 0.2] or [−0.2; 0] are considered as very weak; [0.2–0.5] or [−0.5; −0.2] are considered as weak to mild; up to 0.5 [less than −0.5] is considered as strong to very strong. Agreement between iMg, pMg, and cMg was assessed by agreement rate and concordance with Cohen’s kappa coefficient. Values were considered as “concordant” when iMg and pMg were both “hypo”, “normal” or “hyper”, and “discordant” in all other cases. The results concerning the kappa coefficient were interpreted in relation to the recommendations reported in the literature by Altman [23]: <0.4: no agreement, 0.4–7: poor agreement, >0.7: moderate to strong agreement. To control our samples, we performed the same analysis with iCa and pCa. Association between iMg and other etiologies of de novo AF or hypoMg treatments was assessed using chi^2^ for categorical variables and Student’s t-test or Mann–Whitney test for continuous variables. Lastly, the impact of pH and albumin among discordant Mg, was evaluated with statistical tests. Because of frequent comparison between Mg and Ca physiology, we controlled all our data using iCa and pCa [24,25]. All the tests were two-sided. We defined the *p*-value (type I error) as significant when <0.05. If the *p*-value is <0.001, the results are not precise.

## 3. Results

### 3.1. Characteristic of the Population

Between November 2020 and December 2021, 236 patients with de novo AF were enrolled in the study including 131 from the ED and 105 from the ICUs. Baseline characteristics of de novo AF patients included are presented in Table 1. ED patients were significantly older than ICU patients (76.0 ± 18.1 versus 71.0 ± 11.8). 62.7 % of our population was male, with a higher proportion among ICU patients (70.5 % versus 56.5 %). Hypertension was the main etiology of AF in our population (*n* = 140, 66.5%) with a significantly higher proportion in ED patients (*p* < 0.001). Other etiologies were hyperthyroidism (*n* = 2, 0.9%), hypokalemia (*n* = 35, 15.0%), sepsis (*n* = 36, 15.3%), and dehydration (*n* = 29, 12.3%). Hypokalemia, sepsis, and dehydration were more present in ICU patients (*p* = 0.006, *p* < 0.001, *p* = 0.034 respectively). Regarding hypomagnesemia-inducing medications, the proportion of proton pump inhibitors intake was higher in the ICU (46.7% vs 9.9%, *p* < 0.001). Table 1.

### 3.2. Interpretation of iMg

#### 3.2.1. Reference Values

The reference range values for iMg were determined in the control population constituted in the ED (80 blood samples collected), median iMg was 0.61 mmol/L (95% confidence interval: 0.48–0.65). The reference range was set at 0.48–0.65 mmol/L, while the manufacturer’s reference range was 0.45–0.60 mmol/L.

#### 3.2.2. Correlation and Agreement between iMg and pMg or cMg

**Figure 1:**
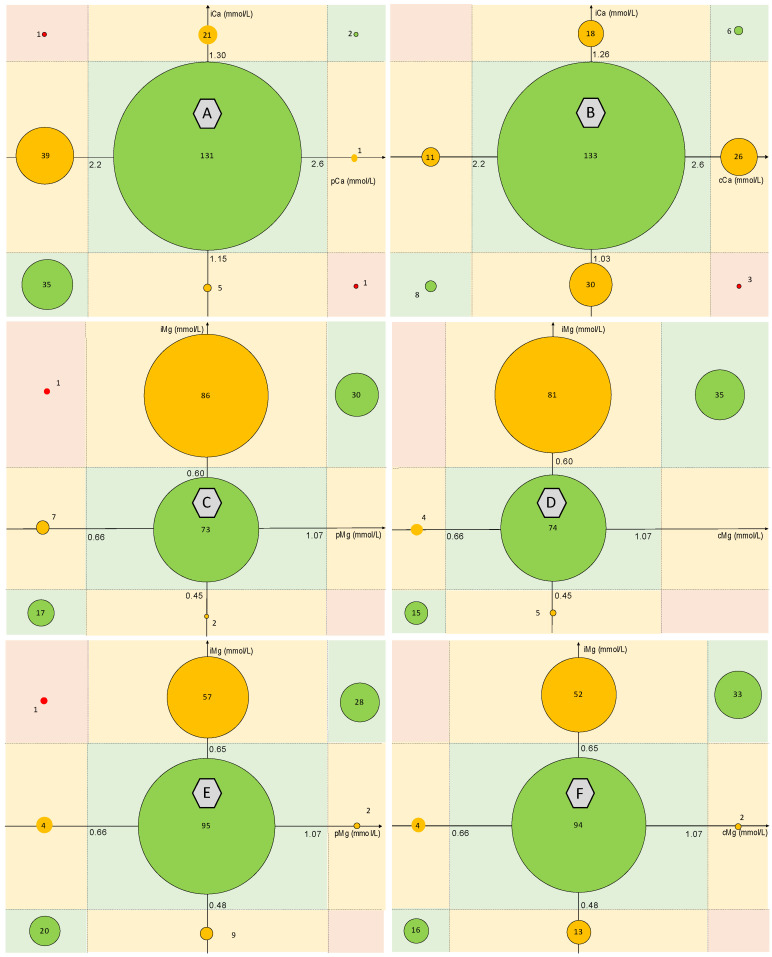
Agreement between the biological interpretation of ionized and total Mg and Ca forms according to the reference values used. A: agreement between iCa and pCa; B: agreement between iCa and cCa; C: agreement between iMg and pMg according to the manufacturer’s range; D: agreement between iMg and cMg using the manufacturer’s range; E: agreement between iMg and pMg using the determined reference range; F: agreement between iMg and cMg using the determined reference range. Correction formulas used for cCa and cMg are: cCa = pCa + 0.025 (40-albumin) and cMg = pMg + 0.005 (40-albumin). ICa = ionized calcium, pCa = plasmatic calcium, cCa = albumin-corrected calcium. iMg = ionized magnesium, pMg = plasmatic magnesium, cMg = albumin-corrected magnesium.

Correlation was assessed for a total of 236 calcium values and 216 magnesium values. Spearman correlation coefficients were 0.85 between iMg and pMg, 0.85 between iMg and cMg, 0.60 between iCa and pCa, and 0.25 between iCa and cCa. Agreement between the biological interpretation of ionized and total Mg and Ca forms according to the reference values used is presented in Figure 1. Agreement between iCa and pCa was 71% with a Κ coefficient at 0.40. It decreased to 63% (Κ = 0.14) between iCa and cCa. Regarding Mg values, using the manufacturer’s ranges, the agreement between iMg and pMg was 56% (Κ = 0.37), and 58% (Κ = 0.39) between iMg and cMg. The agreement increased to 66% (Κ = 0.47) between iMg and pMg, and 67% (Κ = 0.48) between iMg and cMg using the reference values determined in our study (Table 2).

#### 3.2.3. Impact of Albumin and pH

The influence of pH and albumin on the concordance/discordance between ionized and total plasmatic values of Mg and Ca is presented in Table 2. The manufacturer’s reference values for iMg were used for the agreement assessment. A significantly lower albumin median was observed among discordant calcium levels (hypo total plasmatic/normal ionized) compared to concordant calcium levels (normal pCa-normal iCa) (Table 2, *p* < 0.001). This result was not observed for Mg (Table 2, *p* = 0.32). A significantly higher pH median was observed among discordant calcium levels (normal total plasmatic-hyper ionized) compared to concordant calcium levels (normal pCa-normal iCa) (Table 2, *p* = 0.026) with a higher proportion of acidosis in the discordant group (Table 2, *p* = 0.019). This result was not observed for Mg (Table 2, *p* = 0.34 and *p* = 0.37 for pH median and proportion of acidosis respectively).

### 3.3. Incidence of Hypo-iMg among de novo AF

The repartition of hypo-normo and hyper iMg is presented in Table 3. Using the manufacturer’s ranges, 20 (8.5%) of the 236 AF patients were classified in the hypoiMg group, 11 (10.5%) from the ICU, and 9 (6.9%) from the ED. Using the range determined in our study, 30 (12.7%) AF patients were classified in the hypo-iMg group, 18 (17.1%) from the ICU, and 12 (9.2%) from the ED. The proportions of hypo-iMg between AF and control groups (Total, ICU, ED) were not significantly different, except for ED patients, when using the ranges determined in our study (*p* < 0.001). The study of the association between hypo-iMg and the main acute AF etiologies (hypertension, hyperthyroidism, hypokalemia, sepsis, and dehydration) highlighted a significant association between hypo-iMg and hypokalemia (*p* = 0.01). Indeed, hypo-iMg was present in 9 of the 35 patients with hypokalemia (25.7%) and in 21 of the 201 patients without hypokalemia (10.4%).

## 4. Discussion

Ionized Mg is a promising biomarker in emergency medicine considering the importance of its role in cardiac automatism. iMg measurement is available in point of care, and guidelines for sampling, measurement, and result reporting have been issued to ensure the quality of the results [26].

### 4.1. Reference Values

The measurement of iMg in our study was carried out on a Stat Profile^®^ PRIME Plus analyzer from Nova Biomedical. The manufacturer’s reference values (0.45 to 0.60 mmol/L) were determined in 125 healthy volunteers [12]. Although Mg is the fourth most present cation in the human body (second in the intracellular compartment), its measurement (ionized form) is still rare, and the literature is poor regarding reference ranges [27]. In our study, the biological interpretation agreement between iMg and pMg (or cMg) values increased when applying the reference ranges established in our study. Reinforcing the idea that the iMg reference values must be updated. The initial reference interval (95th percentile) used for total serum Mg concentration was 0.75–0.95 mmol/L, it has been determined in 15,820 “healthy normal individuals” aged 18–74 years in 1974 [28]. There is still a debate about those ranges and daily recommended intakes. Indeed, Mg consumption decreased over the last 30 years due to the increase in fast food and the decrease in fruits and vegetables consumption [29]. A recent study suggests a 0.85 mmol/L threshold for the definition of hypomagnesemia, after collecting 43 serum Mg reference range values from 16 countries [30]. The lack of reliable data concerning the interpretation of Mg and iMg values may be related to the fact that Mg measurement is rarely prescribed in routine care, since mild hypomagnesemia rarely has clinical implications. Furthermore, the measurement of iMg requires specialized equipment and is even less used. iMg values may also be subject to individual interferences [1]. Lastly, studies have shown that iMg accounts for 60–64% of total pMg [31,32]. In a study published in 2007, a weak correlation has been described between iMg and pMg, and between iMg and albumin. In the same study, authors set iMg reference range values to 0.48–0.63 mmoL/L [33]. These values are similar to the ones described in our study (0.48–0.65 mmol/L). A large observational study using different measuring instruments would be of value for the harmonization of reference range values allowing an appropriate evaluation of Mg status.

### 4.2. Influencing Variables

The impact of pH and albumin variations on calcium levels has been widely studied [25,34]. Patients with severe hypoalbuminemia present low total calcium levels without clinical relevance since the ionized concentration remains normal [25]. Furthermore, patients with severe acidosis present normal total calcium levels while the ionized (active) form is increased [24]. Since Mg content and physiology are considered analog to that of calcium, pMg and iMg levels are assumed to be similarly affected by pH and albumin variations [9,10]. Our results are in accordance with these observations concerning calcium levels, constituting an external validation criterion. However, we did not find such results for Mg. A strong correlation has been observed between iMg and pMg (r = 0.85), reinforcing the idea of a limited impact of pH and albumin on iMg levels. Furthermore, the albumin correction of Mg values did not improve the correlation coefficient (r = 0.85 between iMg and cMg). This strong correlation observed in our study is inconsistent with the findings of a study in a smaller cohort of 48 ICU and ED patients, where a weak correlation was observed between iMg and pMg (r = 0.59) [33].

### 4.3. Incidence of Hypomagnesemia during de novo AF

To our knowledge, we performed the first study on the incidence of hypomagnesemia in the setting of de novo AF in ED and ICU patients. We found that 8.5% of patients (10.5% of ICU patients and 6.9% of ED patients) with de novo AF had hypo-iMg. This incidence seems independent from AF in ICU patients since we observed a similar proportion of hypo-iMg for ICU patients without AF. However, hypo-iMg is significantly higher in ED patients with AF. We did not find any data in the literature to compare our results. However, it has been described that serum Mg is inversely associated with the risk to develop AF in type 2 diabetes patients during a six-year follow-up study [35]. Among 162 patients, 22 (1.4%) developed AF over a median follow-up of 25.3 months, suggesting an association between hypomagnesemia and AF without a temporal relationship between hypomagnesemia and the early occurrence of AF [36]. Lastly, the Framingham study showed that individuals in the lowest quartile of serum magnesium were 50% more likely to develop AF compared to those in the upper quartiles [37]. iMg during AF was more studied during the peri-operative period. For example, the iMg level immediately before admission to ICU may be associated with the development of post-operative AF after surgery for esophageal cancer [38]. Among ED patients, the 6.9% incidence of hypo-iMg is lower than in the literature. An Australian study showed a 10% of abnormal (hypo and hyper) serum Mg level [39] and a recent study based on 1008 patients found a 17.2% rate of hypomagnesemia [40]. When using the ranges determined in our study, the 9.2% incidence of hypo-iMg seems more relevant especially since it is significantly higher than in the control group. Finally, a profound Mg depletion impacts the homeostasis of potassium and leads to hypokalemia [7]. The significant link between hypo-iMg and hypokalemia highlighted in our study is therefore relevant and minimally places hypomagnesemia as an “etiology of an AF etiology”.

### 4.4. Limitations

Our study has some limitations. Firstly, our monocentric design and the relatively low number of patients included may affect the interpretation of our results. However, the sample size was sufficient for the primary objective of evaluating concordance/correlation. The study could be considered underpowered for secondary objectives, i.e., to determine the factors associated with the observed discrepancies. A multicentric design would increase the power and the generalization of our result.

## 5. Conclusions

Our study highlights the need for more accurate reference range values for iMg. Furthermore, albumin and pH did not impact iMg and pMg interpretation, suggesting that blood Mg content is not identical to that of calcium. Furthermore, the incidence of hypoiMg among de novo AF patients is 8.5% (12.7% within the range defined in our study) in our ICU and ED population. Magnesium therapy has been suggested for a long time in the management of AF [7] and seems very interesting for patients referred to an emergency department [41]. Although we did not find a higher rate of hypoMg among de novo AF in ICU, it seems that ED patients with de novo AF have more hypoMg and should benefit from magnesium therapy.

## Figures and Tables

**Table 1:** Characteristics of the study population (de novo AF patients).

	Total (*n* = 236)	ED (*n* = 131)	ICU (*n* = 105)	*p*
Age, years, mean ± SD	75 ± 15.7	76 ± 18.1	71 ± 11.8	0.001
Sex, *n* (%) male	148 (62.7)	74 (56.5)	74 (70.5)	0.027
BMI, kg/m², mean ± SD	27.6 ± 6.8	27.0 ± 5.9	28.2 ± 7.4	0.37
CHADSVASC, mean ± SD	2.9 ± 1.9	2.2 ± 2.2	2.8 ± 1.6	<0.001
Main acute AF etiologies, *n* (%)				
Hypertension	140 (66.5)	75 (67.2)	65 (65.7)	<0.001
Hyperthyroidism	2 (0.9)	2 (1.5)	0 (0.0)	0.50
Hypokalemia (<3.5 mmol/L)	35 (15.0)	12 (9.2)	23 (22.1)	0.006
Sepsis	36 (15.3)	9 (6.9)	27 (25.7)	<0.001
Dehydration	29 (12.3)	11 (8.4)	18 (17.4)	0.034
Hypomagnesemia inducing-medication intake during diagnosis, *n* (%)				
Proton Pump Inhibitor	62 (26.3)	13 (9.9)	49 (46.7)	<0.001
Diuretics	73 (30.9)	39 (29.8)	34 (32.4)	0.64

ED: emergency department, ICU: intensive care unit, SD: standard deviation, BMI: body mass index, kg: kilogram, m^2^: square meters, CHADSVASC: anticoagulation score.

**Table 2:** Impact of albumin and pH on the interpretation of total plasmatic concentration of 2 divalent cations: Ca and Mg.

Impact of Albumin				
Agreement between Ionized and Total Values		Concordant: Normal Ionized and Total Plasmatic	Discordant: Normal Ionized and Hypo Total Plasmatic	*p*
Calcium	*n*	131	39	/
	Albumin, g/L, median [p25; p75]	36.8 [30.3; 41.4]	28.2 [22.9; 33.3]	<0.001
Magnesium	*n*	73	7	/
	Albumin, g/L, median [p25; p75]	35.1 [30.5; 40.8]	32.7 [21.7; 37.9]	0.32
Impact of pH				
Agreement between ionized and total values		Concordant: normal ionized and total plasmatic	Discordant: hyper ionized and normal total plasmatic	*p*
Calcium	*n*	131	21	/
	pH, median [p25; p75]	7.43 [7.39; 7.46]	7.40 [7.38; 7.42]	0.026
	Acidosis—pH < 7.32, n (%)	2 (1.5)	3 (14.3)	0.019
Magnesium	*n*	73	86	/
	Median [p25; p75]	7.43 [7.39; 7.46]	7.42 [7.39; 7.47]	0.34
	Acidosis—pH < 7.32 *n* (%)	7 (9.6)	5 (5.8)	0.37

SD = standard deviation. g/L = grams per liter. mmol/L = millimole per liter.

**Table 3:** Repartition of hypo, normo, and hyper iMg among 236 patients with de novo AF and controls.

		TOTAL *n* (%)			ICU *n* (%)			ED *n* (%)		
		AF Group (*n* = 236)	Control Group (*n* = 198)	*p*	AF Group (*n* = 105)	Control Group (*n* = 118)	*p*	AF Group (*n* = 131)	Control Group (*n* = 80)	*p*
Manufacturer’s range of iMg (mmol/L)	<0.45	20 (8.5)	15 (7.6)	0.93	11 (10.5)	14 (11.9)	0.93	9 (6.9)	1 (1.3)	0.09
	0.45 to 0.60	89 (37.7)	77 (38.9)		32 (30.5)	34 (28.8)		57 (43.5)	43 (53.8)	
	>0.60	127 (53.8)	106 (53.5)		62 (59.0)	70 (59.3)		65 (49.6)	36 (45.0)	
IMAF’s range of iMg (mmol/L)	<0.48	30 (12.7)	21 (10.6)	0.01	18 (17.1)	17 (14.4)	0.40	12 (9.2)	4 (5.0)	<0.001
	0.48 to 0.65	115 (48.7)	124 (62.6)		37 (35.2)	52 (44.1)		78 (59.5)	72 (90.0)	
	>0.65	91 (38.6)	53 (26.8)		50 (47.6)	49 (41.5)		41 (31.3)	4 (5.0)	

Values are expressed as number of patients (percentages) for all patients, ED patients and ICU patients using manufacturer’s range values in the upper part and our ranges in the lower part. ED = emergency department, ICU = intensive care unit; AF = atrial fibrillation. iMg values are expressed in mmol/L. *p*-value is considered significant if <0.05.

## Data Availability

All relevant data are presented in this manuscript.
